# Supplementary figures and images for: Thermal biology of two tropical lizards from the Ecuadorian Andes and their vulnerability to climate change
Source: PLoS One. 2020 Jan 24;15(1):e0228043. doi: 10.1371/journal.pone.0228043 (PMC6980609; doi:10.1371/journal.pone.0228043)

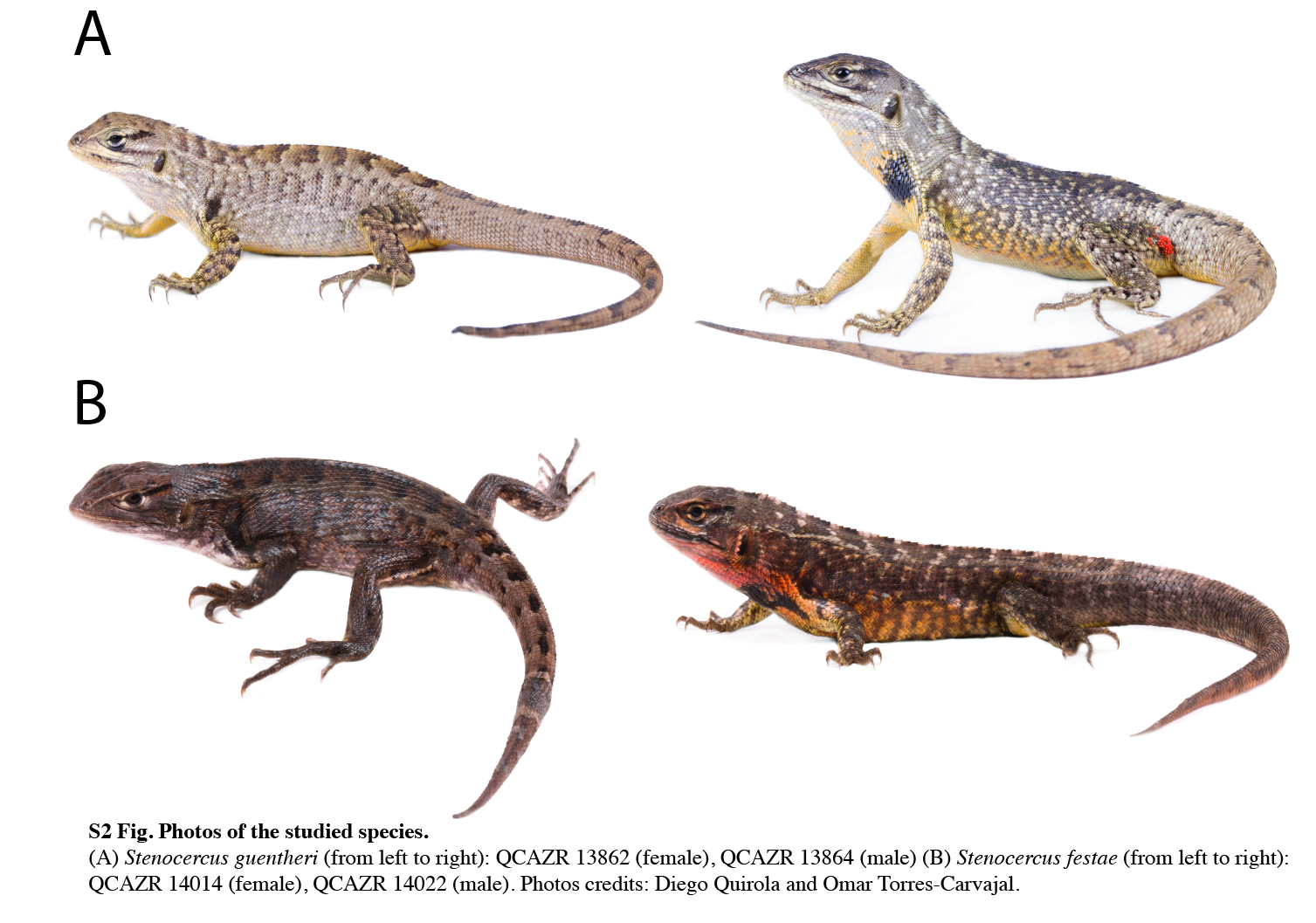

Supplement: S1 Fig — (A) Stenocercus guentheri (from left to right): QCAZR 13862 (female), QCAZR 13864 (male) (B) Stenocercus festae (from left to right): QCAZR 14014 (female), QCAZR 14022 (male). (TIF) [file pone.0228043.s001.tif]

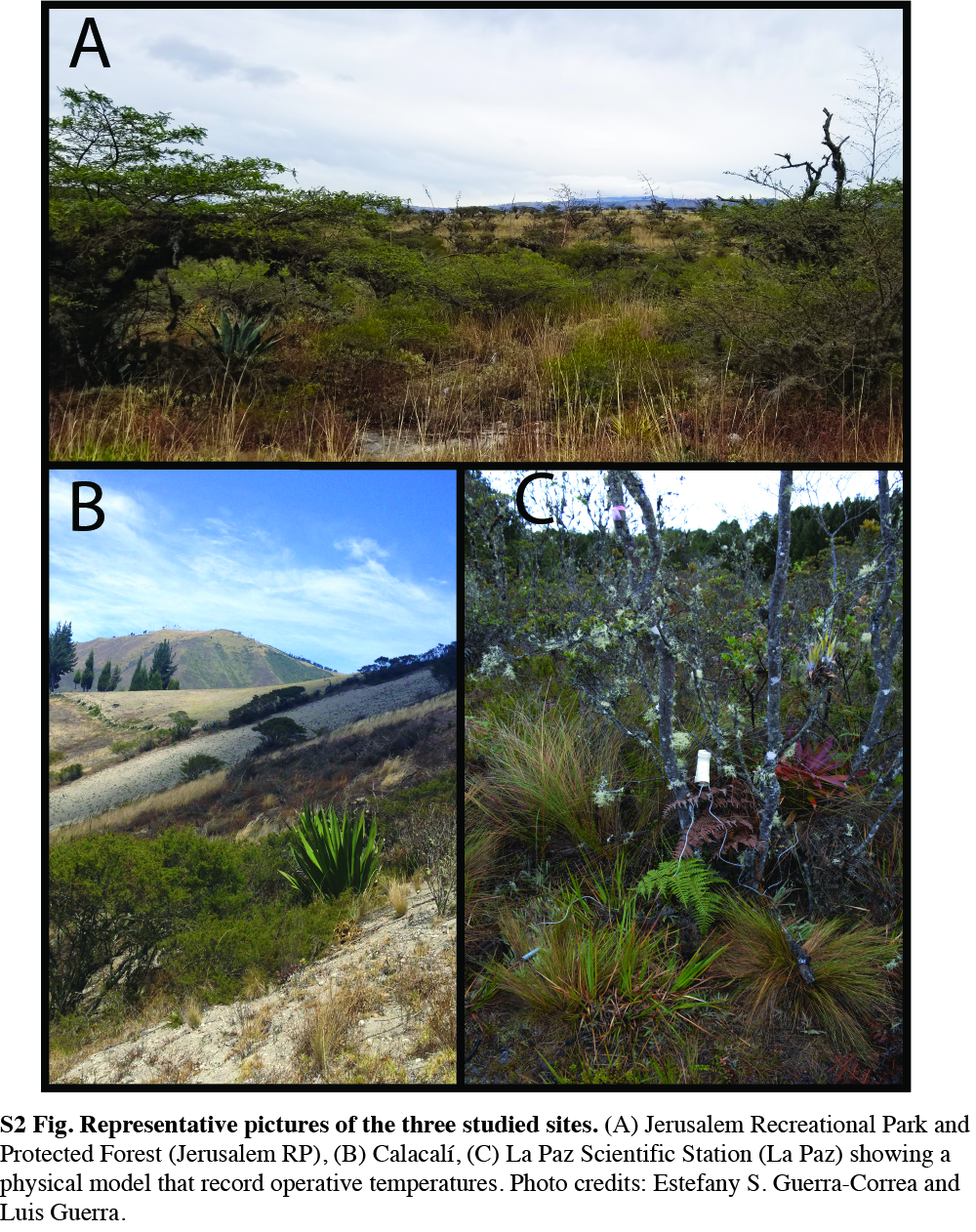

Supplement: S2 Fig — (A) Jerusalem Recreational Park and Protected Forest (Jerusalem RP), (B) Calacalí, (C) La Paz Scientific Station (La Paz) showing a physical model that record operative temperatures. (TIF) [file pone.0228043.s002.tif]
