# Supplementary material for: Thermal biology of two tropical lizards from the Ecuadorian Andes and their vulnerability to climate change
Source: PLoS One. 2020 Jan 24;15(1):e0228043. doi: 10.1371/journal.pone.0228043 (PMC6980609; doi:10.1371/journal.pone.0228043)
Supplement: Attachment — Submitted filename: Rebuttal letter.docx [file pone.0228043.s003.docx]

October 21, 2019

Dear Academic Editor,

This rebuttal letter outlines our point-by-point responses to the comments made on the MS ID **PONE-D-18-24241R1**, submitted previously for consideration to PLOS ONE Journal. We thank you and the reviewer for the detailed and constructive critiques. As you suggested changes have been made according to the limitations and restrictions of the models applied in this study. Also, we added supplementary material with photos of the studied species and their microhabitats, and English language has been reviewed as you suggested. We hope that this new version will be suitable for publication in PLOS ONE. Please let us know if we can offer any additional clarification or respond to any additional concerns.

Sincerely,

Estefany S. Guerra-Correa & co-authors

Reviewer’s comments are in blue, the author’s responses are in black. Each comment is numbered. Revised text is in red with reference to page (P) and line numbers (L) in bold between parentheses.

1) 27-29. Give a justification that makes the reader understand the objectives of this study.

We changed this part as follows:

**(P 2: L 27-32)** This study aims to analyze the thermal biology and climatic vulnerability of two closely related lizard species (*Stenocercus festae* and *S. guentheri*) inhabiting the Ecuadorian Andes at high altitudes. We present a comparative analysis of four physiological parameters— body temperature (*T*_b_), preferred temperature (*T*_pref_), critical thermal maximum (CT_max_), and critical thermal minimum (CT_min_)— to evaluate the variation of thermophysiological traits among these populations that inhabit different environmental and altitudinal conditions.

2) 43-45. This conclusion does not follow logically from the results.

We agree. We have modified this part as follows:

**(P 2: L 43-45)** We suggest that studies on the thermal biology of tropical Andean lizards living at high altitudes are extremely important as these environments exhibit a unique diversity of microclimates, which consequently result on particular thermophysiological adaptations.

3) 70. Having less option for thermoregulation because they have evolved low thermal tolerance.

**(P 3: L 67-71)** For instance, some tropical and subtropical forest lizards are facing more challenges to withstand short-term temperature shifts because they live in habitats where operative temperatures are relatively less variable; having less options for thermoregulation as they have evolved narrower thermal tolerance in contrast to species of temperate zones.

4) 80-81. Do you really believe this? The existence of large spatial ranges in microenvironmental temperatures at mountains is long known (ex. see Geiger 1967). It comes from increased radiation combined with the adiabatic gradient in air temperatures. Sinervo’s models do not include such ranges, and thus assume lizards cannot just go to the shade and keep eating from there.

I understand the need to show relevance for this study, but it feels wrong that one has to look away from reality and literature (i.e. the existing and long known spatial ranges in temperatures at open habitats in the mountains and the ability of lizards and most ectotherms to thermoregulate behaviorally) in order to believe that lizards will be extinct.

I’d like this study to be published. I think it is relevant, but that one argument is really hard to accept. I see it comes from a published article, but it does not solve the problem with Sinervos’ models.

We believe there are some important factors which Sinervo’s model do not considered, as the one you pointed out here. We accepted your comment and decide to rewrite this part as follows:

**(P 4: L 77-81)** As a matter of fact, the Intergovernmental Panel on Climate Change (IPCC) have predicted that Andean regional temperature will increase from 1.7 °C to 6.7 °C by 2100 [17]. These radical changes on temperature can provoke negative consequences on lizards since behavioral adjustments will probably not be enough to avoid overheating and consequently life history aspects could be compromised [18].

5) 104-105. I did not see this in the discussion, nor I feel it is expected, since this study is not a comparison of approaches, is it?

No, this study is not a comparison of approaches that’s why we deleted this sentence as you suggested.

6) 119. “..air temperature..”, temperature ranges would be much more informative.

Air temperature ranges are now described for each of the studied sites.

**(P 5: L 116-117)** … where the average yearly temperature is about 16 °C, ranging from an average minimum of 10 °C to a maximum of 24 °C [32, 33].

**(P 5: L 119-120)** At this site, the annual mean air temperature is 13.6 °C, ranging from an average minimum of 7 °C to a maximum of 20 °C [33].

**(P 6: L 128-130)** This site is characterized by fog and cold winds, where the annual mean air temperature is 11°C, ranging from an average minimum of 6 °C to a maximum of 15 °C [33, 36].

7) 120. It would be important to have pictures representative of the species and the habitats/microhabitats studied

Supplementary figures (S1 and S2) showed pictures of the studied species and the microhabitats.

8) 137, is an animal hiding in a crevice inactive? Or simply exhibiting one more of the range of body temperatures available for the species?

We measured the body temperature of active individuals, which were outside their retreats. Most of our body temperature data was influenced by solar radiation and that’s why our Tb values are higher than Tpref values.

9) 147. Data and methods showing the calibration of the body temperatures according to its correlation with the PVC model is needed herein. Authors of ref 17 probably did it, but did they correct dataloggers data accordingly to their correlation with body temperatures?

We clarify this point with details of the calibration of the model and lizards’ body temperature.

**(P 7: L 153-158)** To determine if these models represent the body temperature of a lizard, we registered simultaneously temperature data of the models and body temperature of lizards in the field. Then, we conducted a two-sample t-test that showed there were no significant differences between the mean body temperature of lizards (mean = 29.6 °C, SD = 0.74) and the mean temperature of the models (mean = 30.1 °C, SD = 1.37); N= 27, t= -1.695, *P* = 0.097.

10) 142. “Represents” instead of “explores”. The original definition from Bakken (the force of environmental temperatures to deviate an animal from its preferred temperature differs from the typical use (the set of body temperatures that a lizard exhibits). Which one are you using here?

We clarified this part as follows:

**(P 6: L 144-146)** To estimate the operative temperature (*T*_e_), defined by Bakken [38] as a thermal index that represents the set of body temperatures a lizard experiences at it’s spatial scale, we used physical models that simulate a lizard’s phenotype in size and color [39, 40, 41].

11) 151. Which ones? Did they represent the whole range of operative temperatures available for the lizards?

Physical models where placed at two completely different sites as described: **(P 7: L 151-153)** One model was placed over the ground at shaded refuge sites (e.g. under small bushes, rocks or logs) and the other model registered open sites exposed to direct sunlight, as bare rocks and logs where active lizards were observed (S2C Fig). For more detail of the type of microhabitat chosen, you can refer to supplementary figure 2C as stated above.

Models were placed on randomly chosen sites where lizards were observed. These sites represent 4 different microhabitats, which estimate the range of operative temperatures these lizards exhibit.

12) 166. The dates for which air temperatures were taken need to be described.

Dates for air temperature are now included as follows:

**(P 7: L 170-173)** Owing to logistics, we obtained *T*_air_ data for 3 months in Calacalí (between 5 May and 17 July 2015) and Jerusalem RP (between 28 September 2017 and 28 November 2017), and 10 months in La Paz (between 6 October 2014 and 22 October 2015; excluding January and September 2015).

13) 177-179. At which temperature? Or a thermal gradient was available for them to thermoregulate?

We kept them at room temperature.

14) 228-229. But Tpref it most often subestimates Topt. Thus giving a sense of more danger for lizards. This paper provides a better theoretical background for justifying the use of Tpref

[https://www.journals.uchicago.edu/doi/abs/10.1086/527502](https://www.journals.uchicago.edu/doi/abs/10.1086/527502)
Still, it is unclear what happens with lizards if the min temp available reaches the Tpref (that they feel good at the shade but hot at the sun?).

First of all, thanks for the reference you shared with us, it is now cited to justify using Tpref in these analyses. Secondly, we agree with you and changed the analyses’ inputs as follows: **(P 10: L 232-234)** TSM is the difference between a lizard’s optimal temperature (*T*_opt_) and the mean hourly minimal *T*_e_ in the shade [*T*_e_s min; 3].

15) 230-231. This is an incorrect interpretation of ref 15. Sorry but, logically, the minimum values across shaded models placed at one site give the total thermoregulation scope for the population living there, the mean across them, the average scope. The maximum registered temperature in the shade certainly helps overestimating vulnerability to climate warming. Also, if you are using dataloggers temperatures to measure temperatures in the shade, why do you need air temperatures for, and vice versa? You need to understand what each variable truly represents for the lizards and then choose what to use.

We agree, we modified this part as follows:

**(P 10: L 236-238)** For the latter analyses, we used the minimum values of *T*_e_ of models exposed to shade sites because they are better descriptors of the thermal microenvironment these lizards have in order to avoid warming [15].

In these analyses we used the operative temperatures registered by the physical models because they describe more precisely the microhabitat temperature. Air temperatures are essential to show the environmental differences of each site.

16) 244. Which ones? You cite two different descriptors.

We don’t understand what you meant with this comment.

17) 261. Is this the mean for the species or for one of the individuals of that species?

That value corresponds to the mean for the species.

18) 277-278. Describe the hypothesis tested herein.

This part was restructured as follows:

**(P 13-14: L 308-313)** Phylogenetic and environmental adaptive forces seem to be responsible for shaping the thermal biology of other lizard taxa, such as *Liolaemus* [23], a lineage suggested to exhibit adaptive radiation parallel to *Stenocercus* on different areas of South America (i.e. similar phenotypic variation patterns) [45]. As we hypothesize, there are significant inter- and intraspecific differences in some thermophysiological traits between the three populations studied herein.

19) 297. You need to be much more precise here. There are multiple facets of climate warming and only some are of interest for this study.

We moved this part to the end of the discussion.

20) 306-310. Please explain more clearly these differences, making explicit the altitude of each site and hints of the direction of change in thermal tolerance with altitude and scope for intraspecific variation, there is relevant literature on these topics that you can use.

We added the altitude of each site in this section:

**(P 14: L 317-320)** This suggests that some differences in physiological traits among populations/species of *Stenocercus* are caused by climatic factors that change systematically along altitude (La Paz 3100 m, Calacalí 2950 m, and Jerusalem RP 2278 m), creating selective pressures on physiology [46, 47].

21) 324. If you think there were biases during the measurement of thermophysiological data, it is honest to point them out and identify how to correctly interpret the results.

We clarified this idea as follows:

**(P 15: L 334-338)** On the other hand, *T*_pref_ experiments were performed along the day from 9:00, covering hours at which lizards were not found basking to direct sunlight in the field. Thus, is important to consider that probably these lizards are more used to perform activities at low temperatures given that the mean air temperature is 13.7 ºC and 10.9 ºC for Calacalí and La Paz, respectively.

22) 347. But variable across their range, nonetheless...and in the end, is this prediction necessary? I would simply delete it.

We do consider necessary this prediction as we can show the reader which of the three populations studied herein will be more vulnerable to climate change.

23) 350-351. But because the models used in 17 throw away all the thermal variation you describe herein, by taking means of operative temperatures and comparing them with mean Tpref...

This section was deleted.

24) Fig.1 If you end up really using air temperatures for your calculations, I think that would go better in a supporting material file, together with info on how the model calibration was done with a living animal, and how the temperatures registered by the model were corrected accordingly to the correlation.

If the Fig is kept herein, better resolution is needed, and also adequate proportions of line/text for its publication.

We decided to keep this figure (not as supplementary); resolution is now improved. Details of how the model was calibrated are described on Specimen and field data collection section.

25) Fig. 2. This graph is probably the only one you need, plus pics of the animals and habitats/microhabitats. This graph needs better resolution and it probably makes much more sense after you represent Tpref as a band (ex. range of Tpref=Vtmax-Vtmin). That band should include your field Tb. If field Tb are above the Tpref band of the same individuals then something is wrong. It could be with the measures in the thermal gradient, or the sun was hitting the thermocouple during the measurements of Tb, or the thermocouple was still warm from the hand or backpack… If so, I am afraid the data is not publishable.

The resolution of this figure improved. We kept this figure as the original because we want to show the time of capture of the lizards and the corresponding body temperature and the mean Tpref value of each species and study site. We explained this point in the discussion section as follows: **(P 14: L 327-335)** Two populations in this study show higher *T*_b_ than *T*_pref_, (Table 1). On one hand, high *T*_b_ values are explained by the hours of capture of the individuals, which for most of the lizards assessed in this study correspond to hottest time of the day. Besides, Pearson [49] has shown that tropical lizards living at high altitude can achieve high body temperatures by using solar radiation. On the other hand, *T*_pref_ experiments were performed along the day from 9:00, covering hours at which lizards were not found basking to direct sunlight in the field. Thus, is important to consider that probably these lizards are more used to perform activities at low temperatures given that the mean air temperature is 13.7 ºC and 10.9 ºC for Calacalí and La Paz, respectively.
